# Transferrin-Functionalized Liposomes for the Delivery of Gallic Acid: A Therapeutic Approach for Alzheimer’s Disease

**DOI:** 10.3390/pharmaceutics14102163

**Published:** 2022-10-11

**Authors:** Stéphanie Andrade, Joana A. Loureiro, Maria C. Pereira

**Affiliations:** 1 LEPABE—Laboratory for Process Engineering, Environment, Biotechnology and Energy, Faculty of Engineering, University of Porto, Rua Dr. Roberto Frias, 4200-465 Porto, Portugal; 2 ALiCE—Associate Laboratory in Chemical Engineering, Faculty of Engineering, University of Porto, Rua Dr. Roberto Frias, 4200-465 Porto, Portugal

**Keywords:** neurodegeneration, amyloid β peptide, aggregation, fibril, nanoparticle, PEGylated liposome, stealth liposome, drug delivery system, blood–brain barrier, targeting, transferrin receptor

## Abstract

Senile plaques composed of amyloid β (Aβ) fibrils are considered the leading cause of Alzheimer’s disease (AD). Molecules with the ability to inhibit Aβ aggregation and/or promote Aβ clearance are thus a promising approach for AD therapy. Our group recently demonstrated that gallic acid (GA) has strong anti-amyloidogenic properties. In this study, stealth liposomes were prepared for the delivery of GA for AD therapy. The liposomes were functionalized with transferrin (Tf) to direct them to the brain, since Tf receptors are overexpressed in the endothelial cells of the blood–brain barrier. GA-loaded Tf-functionalized liposomes showed mean diameters of 130 nm, low polydispersity index values, and neutral zeta potential. Moreover, the produced nanocarriers promoted the sustained release of GA over 5 days and are physically stable for 1 month under storage conditions. Furthermore, GA-loaded Tf-functionalized liposomes showed a strong ability to interact with Aβ_1-42_ monomers, slowing down the Aβ monomer-to-oligomer and oligomer-to-fibril transitions and decreasing the number of fibrils formed by 56%. In addition, the NPs disaggregated approximately 30% of preformed Aβ fibrils. The presented results suggest that Tf-functionalized liposomes could be a viable platform for the brain delivery of GA for AD therapy. Studies with animal models of AD will be valuable for validating the therapeutic efficacy of this novel liposomal formulation.

## 1. Introduction

Alzheimer’s disease (AD) is a progressive neurological condition and the world’s most common form of dementia. AD patients face memory problems and other cognitive impairments caused by brain atrophy and death of brain cells [1]. Microscopically, the brain of AD patients is characterized by two central cardinal lesions—extracellular senile plaques and intracellular neurofibrillary tangles. While senile plaques comprise amyloid-β (Aβ) peptides aggregated into fibrils, neurofibrillary tangles are composed of hyperphosphorylated tau protein [2]. A growing body of research supports the idea that Aβ aggregation and decreased Aβ clearance are the leading causes of AD onset.

As the world’s population ages, AD is becoming more prevalent, placing pressure on patients and their families. The four medications currently accessible for AD therapy—donepezil, rivastigmine, galantamine, and memantine—are merely palliative [3]. Hence, there is an urgent need to develop treatments for AD that can prevent, stop the progression of, delay, or even reverse the disease. To date, hundreds of natural and synthetic molecules with the ability to prevent Aβ aggregation and help in Aβ clearance have been identified, bringing hope for the treatment of AD. Gallic acid (GA), a polyphenolic compound easily found in blueberries, strawberries, plums, grapes, and so on, has aroused great interest in AD due to its antioxidant, anti-inflammatory, and anti-amyloidogenic properties [4].

Various innovative strategies have been established for drug delivery. Encapsulating molecules into nanoparticles (NPs) allows for protecting them from degradation and transporting them by hiding their physicochemical properties [5]. Among the NPs employed in biomedical applications, liposomes are one of the most widely used and secure [6]. Liposomes are spherical vesicles composed of natural or synthetic lipids arranged in bilayers, forming aqueous compartments. This structure allows the encapsulation of hydrophilic molecules in the aqueous core, or hydrophobic ones in the lipid bilayer. Moreover, liposomes are recognized for their biocompatibility, biodegradability, high stability, and low toxicity [7]. Conventional liposomes composed of neutral and/or anionic lipids were the first generation of lipid vesicles created and used in the pharmaceutical industry [8]. However, their rapid uptake by the mononuclear phagocyte system (MPS) after systemic administration represents the major drawback to their clinical application. It was in this context that long-circulating liposomes emerged. To increase the liposomes’ stability and blood circulation time, sterically stabilized liposomes were introduced by adding a coating to the liposomes’ surface. The presence of the synthetic polymer poly-(ethylene glycol) (PEG) on the liposomes’ surface (stealth liposomes) has been shown to extend blood circulation time while reducing MPS uptake by inhibiting electrostatic and hydrophobic interactions between liposomes and serum components [9].

Due to the blood–brain barrier (BBB), drug delivery to the brain has been one of the most challenging issues that researchers face in developing effective treatments for brain illnesses. BBB is an organized interface between the peripheral circulation and the central nervous system (CNS) that acts as a barrier protecting the CNS from possible chemical damage [10]. If, on the one hand, the BBB blocks the transport of potentially toxic or harmful substances from the bloodstream to the brain, on the other hand, it prevents the entry of therapeutics. Thus, approaches combining NPs with receptor-mediated transcytosis (RMT) have been investigated as a way to get across the BBB and so increase the effectiveness of brain delivery. This strategy is based on the specific interaction of ligands present in the NPs’ surface with receptors overexpressed on the luminal side of endothelial cells of the BBB [11]. The transferrin receptor (TfR) is currently the most widely explored receptor for BBB targeting [12]. Besides the BBB, TfRs are likewise highly expressed on the plasma membrane of neurons [13]. The efficacy of liposome functionalization with Tf in targeting the BBB and increasing the NPs’ penetration across the BBB via the RMT pathway has already been intensively demonstrated in in vitro BBB models as well as animal models [2,14,15,16]. Moreover, Chen et al. (2016) demonstrated that Tf-modified liposomes cross the BBB without compromising their structure [17]. As a result, this strategy has proven to increase the amount of drugs in the brain [18,19].

The present study aims to develop Tf-functionalized stealth liposomes loaded with GA for AD therapy. The produced liposomes were characterized in terms of hydrodynamic diameter, polydispersity index (PdI), zeta potential, encapsulation efficiency (EE), loading capacity (LC), GA release profile, and physical stability under storage conditions. Furthermore, the therapeutic efficiency of the optimized drug delivery system (DDS) for AD prevention and treatment was evaluated by studying the interaction of the nanosystem with the Aβ_1-42_ peptide. This is the first DDS using Tf for the targeted delivery of GA to date.

## 2. Materials and Methods

### 2.1. Materials

1,2-distearoyl-sn-glycero-3-phosphocholine (DSPC, MW 790), cholesterol (CHOL, ovine wool, MW 387), 1,2-distearoyl-sn-glycero-3-phosphoethanolamine-N-[methoxy(polyethylene glycol)-2000] (18:0 PEG2000 PE, MW 2805), and 1,2-distearoyl-sn-glycero-3-phosphoethanolamine-N-[amino(polyethylene glycol)-2000] (DSPE-PEG2000 amine, MW 2791) were purchased from Avanti Polar Lipids Inc. (Alabaster, AL, USA). GA (MW 170), holo-Tf human (MW 80,000), N-(3-dimethylaminopropyl)-N′-ethylcarbodiimide hydrochloride (EDC, MW 192), thioflavin T (ThT, MW 319), chloroform (MW 119), and 1,1,1,3,3,3-hexafluoro-2-propanol (HFIP, MW 168) were obtained from Sigma-Aldrich (St. Louis, MO, USA). Human amyloid-β peptide with 42 amino acid residues (Aβ_1-42_, MW 4514) was acquired from GenScript Biotech (Piscataway, NJ, USA). Uranyl acetate dihydrate (MW 424) was purchased from Electron Microscopy Sciences (Hatfield, PA, USA). Diethyl ether (MW 74) was obtained from José Manuel Gomes dos Santos, Lda (Odivelas, Portugal), while methanol (MW 32) and ethanol absolute (MW 46) were bought from VWR International (Radnor, PA, USA). VWR International also provided phosphate-buffered saline (PBS) tablets (pH 7.4, 10 mM phosphate buffer, 2.7 mM potassium chloride, and 137 mM sodium chloride). PBS was prepared using ultrapure water (Milli-Q Academic, Millipore, Burlington, MA, USA).

### 2.2. Preparation of GA-Loaded Liposomes

DSPC, CHOL, 18:0 PEG2000 PE, and DSPE-PEG2000 amine in a molar ratio of 52:45:3:0.06 were used to prepare GA-loaded liposomes. After conducting preliminary assays, the concentrations of liposomes and GA were fixed at 16.0 mM and 3.6 mM, respectively. The methods used to produce the liposomes included lipid film hydration, reverse-phase evaporation, ethanol injection and ethanol permeabilization. Bare liposomes were also prepared as a control.

#### 2.2.1. Lipid Film Hydration

DSPC, CHOL, 18:0 PEG2000 PE, and DSPE-PEG2000 amine in chloroform were mixed in a threaded glass tube. A thin lipid film was created by manual rotary evaporation using a nitrogen stream. The lipid film was hydrated with 0.600 mL of PBS or a solution of GA in PBS at 3.6 mM. The glass tube was vigorously vortexed for 10 min to create multilamellar vesicles. To reduce the size and lamellarity of liposomes, and obtain large unilamellar vesicles, the liposomal suspension was sonicated using an ultrasonic processor UP400S (40% amplitude; 24 kHz ultrasonic frequency) (Hielscher, Teltow, Germany) in an ice bath for 40 min, with a sequence of 1 min of sonication and 1 min of rest.

#### 2.2.2. Reverse-Phase Evaporation

DSPC, CHOL, 18:0 PEG2000 PE, and DSPE-PEG2000 amine were dissolved in 0.650 mL of diethyl ether:methanol (12:1 *v*/*v*). Dropwise additions of 0.240 mL of PBS or a solution of GA in PBS at 9.0 mM were made to the organic phase. A homogenous opalescent dispersion was produced after the obtained two-phase system was subjected to sonication in a cold-water bath (ultrasonic cleaner, VWR, Radnor, PA, USA) for 5 min. The organic solvent in the mixture was evaporated under continuous magnetic stirring for 2 h at room temperature, producing a viscous gel. PBS was added to complete a final volume of 0.600 mL. The size and lamellarity of the liposomes were decreased, as previously mentioned.

#### 2.2.3. Ethanol Injection

DSPC, CHOL, 18:0 PEG2000 PE, and DSPE-PEG2000 in absolute ethanol were added dropwise (0.120 mL) into 0.600 mL of PBS or a solution of GA in PBS at 3.6 mM under magnetic stirring at 350 rpm and room temperature for 2 h. Sonication was used to reduce the size and lamellarity of the liposomes.

#### 2.2.4. Ethanol Permeabilization

The above-mentioned lipid film hydration method followed by sonication was used to produce liposomes. A solution of GA in PBS at 8.4 mM or PBS was added dropwise to liposomes (16 mM) (30% *v*/*v*). To improve the lipid bilayer’s permeability, the sample was incubated for 1 h at 37 °C with constant agitation (350 rpm). Ethanol was then removed by continuous magnetic stirring at 350 rpm and room temperature for 2 h.

### 2.3. Determination of Entrapment Efficiency and Loading Capacity

The entrapment efficiency (EE) and loading capacity (LC) were determined using the ultracentrifugation technique. A quantity of 0.500 mL of GA-loaded liposomes were placed into centrifugal filters (Amicon^®^, 3 kDa, Merck, Darmstadt, Germany) and subjected to two cycles of centrifugation at 14,500 rpm at room temperature for 30 min (MiniSpin^®^ plus, Eppendorf, Hamburg, Germany). GA-loaded NPs in the concentrated solute were stored at 4 °C for further analysis. Free GA present in the filtrates was quantified by UV−Vis spectroscopy (Synergy 2 Multi-Mode Microplate Reader, BioTek Instruments, Winooski, VT, USA). The measured absorbance at 258 nm was correlated to the GA’s calibration curve. EE and LC were determined using Equations (1) and (2), respectively:

(1) $$\mathrm{EE}\;(\%)=\frac{\mathrm{Total}\;\mathrm{amount}\;\mathrm{of}\;\mathrm{GA}\;-\;\mathrm{amount}\;\mathrm{of}\;\mathrm{free}\;\mathrm{GA}}{\mathrm{Total}\;\mathrm{amount}\;\mathrm{of}\;\mathrm{GA}}\;\times \;100$$

(2) $$\mathrm{LC}\;(\%)=\frac{\mathrm{Total}\;\mathrm{amount}\;\mathrm{of}\;\mathrm{GA}\;-\;\mathrm{amount}\;\mathrm{of}\;\mathrm{free}\;\mathrm{GA}}{\mathrm{Total}\;\mathrm{amount}\;\mathrm{of}\;\mathrm{lipids}}\;\times \;100$$

### 2.4. Functionalization of Liposomes with Transferrin

The NPs’ surface was functionalized with Tf, as described by Neves et al. [20], with slight modifications. The covalent coupling of the DSPE-PEG2000’s amino group with the Tf’s carboxyl group was achieved through a carbodiimide-coupling reaction using EDC as a coupling agent. Here, EDC was added to a solution of Tf in PBS (21.0 µM) at 100× molar excess. The mixture was stirred for 30 min at room temperature at 500 rpm to activate the Tf’s carboxyl groups. The ultracentrifugation technique was then used to separate free EDC from Tf-EDC. Of the mixture, 0.500 mL was placed into centrifugal filters (Amicon^®^, 30 kDa, Merck, Germany) and subjected to two cycles of centrifugation at 14,500 rpm at room temperature for 30 min (MiniSpin^®^ plus, Eppendorf, Germany). Of the activated Tf solution at 21 µM, 0.250 mL was mixed with unloaded or GA-loaded liposomes at 16 mM. Samples were incubated under constant agitation (500 rpm) at 4 °C for 1 h to complete liposome functionalization.

### 2.5. Determination of Transferrin Conjugation Efficiency

The conjugation efficiency (CE) of Tf to the liposomes was determined using the ultracentrifugation technique to separate unbound Tf from Tf-functionalized liposomes, as described by AlSawaftah and colleagues [21]. A quantity of 0.500 mL of Tf-functionalized liposomes was placed into centrifugal filters (Amicon^®^, 100 kDa, Merck, Germany) and subjected to two cycles of centrifugation at 14,500 rpm and room temperature for 30 min (MiniSpin^®^ plus, Eppendorf, Germany). Tf-functionalized liposomes in the concentrated solute were stored at 4 °C for further analysis. Free Tf present in the filtrates was quantified by UV−Vis spectroscopy (Synergy 2 Multi-Mode Microplate Reader, BioTek Instruments, USA) using the Pierce^TM^ bicinchoninic acid (BCA) Protein Assay Kit (Thermo Fisher Scientific, Waltham, MA, USA). Briefly, free Tf was mixed with the working reagent in a 96-well plate. The plate was sealed and incubated at 37 °C for 30 min under constant medium agitation. The measured absorbance at 560 mm was correlated to the Tf’s calibration curve. The CE was determined using Equation (3):

(3) $$\mathrm{CE}\;(\%)=\frac{\mathrm{Total}\;\mathrm{amount}\;\mathrm{of}\;\mathrm{Tf}\;-\;\mathrm{amount}\;\mathrm{of}\;\mathrm{free}\;\mathrm{Tf}}{\mathrm{Total}\;\mathrm{amount}\;\mathrm{of}\;\mathrm{Tf}}\;\times \;100$$

The number of lipids per liposome (N), which was established by Equation (4), was used to estimate the number of transferrin molecules per liposome:

(4) $$N=\frac{4\;\pi \;(r{)}^{2}+\;4\;\pi \;(r-h{)}^{2}}{a},$$

where *r* is the radius of the vesicle, *h* is the thickness of the lipid bilayer, and a is the lipid head group area.

### 2.6. Physicochemical Characterization of Liposomes

#### 2.6.1. Dynamic Light Scattering

Dynamic light scattering was employed to measure the average size and polydispersity index (PdI) of the liposomes (16.0 mM) using a ZetaSizer Nano ZS (Malvern Instruments, Malvern, UK) at a fixed backscattering angle of 173°. Measurements were performed at 25 °C, setting the water’s viscosity, water’s refractive index, lipids’ absorption, and lipids’ refractive index at 0.8872 cP, 1.330, 0, and 1.40, respectively. Data are displayed as size distribution by intensity and result from 3 measurements with 12 runs each.

#### 2.6.2. Electrophoretic Light Scattering

The zeta potential of liposomes (16.0 mM) was assessed by electrophoretic light scattering using a ZetaSizer Nano ZS (Malvern Instruments, UK). Measurements were performed at 25 °C, fixing the parameters as described above. Data result from 3 measurements with 12 runs each.

#### 2.6.3. Attenuated Total Reflection-Fourier Transform Infrared Spectroscopy

Attenuated total reflection-Fourier transform infrared spectroscopy (ATR-FTIR) was applied to validate the functionalization of lipids with Tf. Two μL of unloaded or GA-loaded liposomes, before and after functionalization, were placed on the surface of the FTIR crystal. A nitrogen flow was used to remove the water from the sample, forming a thin film on the crystal’s surface. ATR-FTIR spectra were recorded in absorbance mode using an ALPHA-P spectrophotometer. The scanning range and resolution were set to 375–4000 cm^−1^ and 4 cm^−1^, respectively. Each spectrum was obtained from 64 scans.

### 2.7. Stability of Liposomes at Storage Conditions

The NPs’ physical stability during storage was evaluated for 2 months. Liposomes (16.0 mM) were stored at 4 °C and in the dark. During this period, liposomes’ average size, PdI, and [22] zeta potential were assessed at predetermined time intervals, as previously mentioned. Phenomena of flocculation, creaming, sedimentation, and clarification were also monitored.

### 2.8. In Vitro Release of GA from Transferrin-Functionalized Liposomes

In vitro release studies were conducted using the cellulose dialysis membrane diffusion technique, according to Dejeu’s work [23]. Prior to beginning the assay, cellulose dialysis membranes (Float-A-Lyzer G2, CE, 10 kDa, SpectrumLabs, Los Angeles, CA, USA) were equilibrated at 4 °C with ultrapure water for 12 h, which was replaced with PBS for 1 h. The donor chamber was filled with 1 mL of GA-loaded Tf-functionalized liposomes. The receptor chamber was filled with 5 mL of PBS to mimic physiological conditions. Positive control was prepared by replacing the liposomes with a solution of GA in PBS. The devices were incubated at 37 °C for 5 days while the receptor medium was constantly agitated under magnetic stirring at 250 rpm. A quantity of 0.3 mL of the receptor solution was taken at predetermined time intervals to quantify the amount the GA released. The absorbance of GA at 258 nm was measured by UV−Vis spectroscopy (Synergy 2 Multi-Mode Microplate Reader, BioTek Instruments, USA), and correlated to its calibration curve. The sample was returned to the receptor medium immediately after measurements. GA release profile was plotted according to the following equation:

(5) $$\mathrm{GA}\;\mathrm{released}\;(\%)=\frac{\mathrm{Amount}\;\mathrm{of}\;\mathrm{GA}\;\mathrm{released}\;\mathrm{at}\;\mathrm{time}\;t}{\mathrm{Amount}\;\mathrm{of}\;\mathrm{encapsulated}\;\mathrm{GA}}\;\times \;100$$

### 2.9. Anti-Amyloidogenic Activity of GA-Loaded Transferrin-Functionalized Liposomes

#### 2.9.1. Preparation of Aβ Stock Solutions

Aβ was dissolved in HFIP at 0.5 mg/mL. The sample was stirred (250 rpm) for 3 days at room temperature to disrupt preformed intermolecular H-bonds responsible for Aβ aggregation. To prepare a stock solution of Aβ monomers, HFIP was removed with a nitrogen flow, forming a dried peptide film. PBS was added to the film at a final concentration of 10 μM. The sample was then vortexed and sonicated in a cold-water bath (ultrasonic cleaner, VWR, USA) for 2 min. To prepare a stock solution of Aβ fibrils, Aβ monomers (10 μM) were incubated at 37 °C for 1 h with constant medium agitation. Both stock solutions were immediately used in the ThT fluorescence assays.

#### 2.9.2. Thioflavin T Fluorescence Assay

The kinetic of Aβ aggregation and fibril disaggregation were monitored using a ThT fluorescence protocol adapted from the literature [12]. Aβ monomers or fibrils at 10 μM were mixed with and without GA, unloaded Tf-functionalized liposomes, and GA-loaded Tf-functionalized liposomes in a 96-well plate (black, non-treated, flat bottom, UV-Star^®^). A previously filtered solution of ThT in PBS (0.22 µm) was added to the samples at a final concentration of 50 μM. The final concentration of Aβ and GA in the well was 8 μM and 800 μM, respectively. The microplate was sealed and incubated at 37 °C with continuous medium agitation. ThT fluorescence signal was measured over 5 h using a Synergy™ 2 Multi-Mode Microplate Reader (BioTek Instruments, Winooski, VT, USA) with 420/50 nm and 485/20 nm excitation and emission filters, respectively.

#### 2.9.3. Kinetic Model of Aβ Fibril Formation

After ThT fluorescence measurements, data were normalized and plotted as a function of time (t). A kinetic model for Aβ fibrillation was applied to the data according to Equation (6) [24]:

(6) $$F(t)\;=\;F_{0}\;+\frac{\mathrm{Fmax}}{1+e^{\left[-k\left(t-t_{1/2}\right)\right]}},$$

where F_0_ and F_max_ are the lowest and highest fluorescence values obtained, respectively. k represents the elongation rate constant, and t_1/2_ is the amount of time needed to achieve half of the highest fluorescence value. The fibril growth lag time (t_lag_) was determined based on the last two parameters according to Equation (7) [24]:

(7) $$t_{\mathrm{lag}}=t_{1/2}-\frac{2}{k}$$

#### 2.9.4. Transmission Electron Microscopy

Negative-staining transmission electron microscopy (TEM) samples were prepared as described in a previous study [25]. Of the end-point products from ThT fluorescence experiments, 5 μL was dropped onto a carbon-formvar-coated 400 mesh spacing grid for 5 min and blotted with filter paper to remove the excess sample. A quantity of 5 μL of a uranyl acetate solution in ultra-pure water 2% (*w*/*v*) previously filtered (0.22 µm) and centrifuged at 12,000 rpm for 3 min was placed in the grid for 45 s to stain the samples. The staining solution was blotted with filter paper, and the grid dried. Images were recorded using a JEM 1400 electron microscope (JEOL, Tokyo, Japan) at 80 kV.

### 2.10. Statistical Analysis

Three replicates for each experiment were performed. Results are shown as the average ± standard deviation. Student’s t-tests were performed to determine the statistical significance of the data. Significant differences were defined as *p*-values less than 0.05.

## 3. Results and Discussion

### 3.1. Physicochemical Characterization of Unloaded and GA-Loaded Liposomes

**Figure 1:**
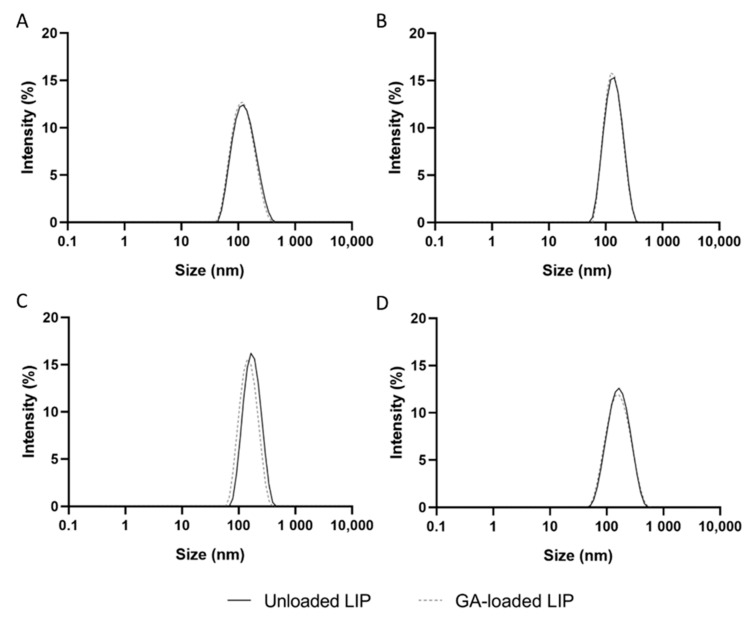
Size distribution by intensity of unloaded (black line) and GA-loaded liposomes (dashed gray line) produced by (**A**) lipid film hydration, (**B**) reverse-phase evaporation, (**C**) ethanol injection and (**D**) ethanol permeabilization.

The physicochemical properties of NPs, such as the hydrodynamic diameter, PdI, zeta potential, EE, and LC, play a significant role in their biological performance. Therefore, unloaded and GA-loaded liposomes were produced using distinct techniques, including lipid film hydration, reverse-phase evaporation, ethanol injection, and ethanol permeabilization, to obtain a formulation with the best physicochemical properties for brain delivery purposes. The liposomes’ composition included DSPC, CHOL, 18:0 PEG2000 PE, and DSPE-PEG2000 amine at a molar ratio of 52:45:3:0.06, which was maintained regardless of the technique since these particular NPs proved to be safe after intravenously administering 10 doses over 3 weeks to adult wild-type mice [26]. Including PEG in the liposomes composition (stealth liposomes) allows them to become invisible to the MPS, thus increasing the blood-circulation time of the lipid vesicles [9]. The physicochemical properties of the unloaded and GA-loaded liposomes produced using the mentioned procedures are presented in Table 1. Figure 1 displays the size distribution by intensity diagrams for each formulation.

The size of a particle is a crucial factor influencing its ability to cross biological barriers, stability, blood-circulation time, drug release, cell uptake, clearance, and toxicity. Evidence demonstrated that NPs larger than 200 nm activate the lymphatic system and are quickly eliminated from blood circulation. Furthermore, NPs larger than 200 nm hardly cross the BBB [27]. The smaller the NP, the easier it is to cross the BBB. However, NPs smaller than 50 nm are related to toxic effects due to the larger surface area-to-volume ratio [28]. Therefore, 50–200 nm has been established as the ideal mean size of an NP for brain delivery.

According to Table 1, all formulations present mean hydrodynamic diameters between 110 and 170 nm, making them appropriate for brain delivery. The technique used to produce liposomes appears to significantly affect their size (*p* < 0.05) in the following order: lipid film hydration < reverse-phase evaporation < ethanol permeabilization < ethanol injection. Furthermore, no significant variations in the NPs’ size were observed following GA encapsulation, regardless of the employed methodology (*p* > 0.05).

Table 1’s comprehensive analysis further demonstrates that all formulations have PdI values below 0.2, indicating that the prepared liposomes have uniform diameters and are, therefore, suitable for drug delivery [29]. Figure 1 confirms that all formulations exhibit a narrow size distribution. No significant differences in PdI values of liposomes were observed regardless of the technique used for the NPs production and GA encapsulation (*p* > 0.05).

The surface charge of an NP is another key physicochemical property affecting its biodistribution, toxicity, and internalization rates [30]. While positively charged NPs have been associated with severe toxicity, negatively charged NPs exhibit lower levels of cellular internalization than cationic and neutral NPs due to the unfavorable electrostatic interactions with cell membranes, also negatively charged [31]. In turn, in addition to crossing the BBB without compromising its integrity, neutral NPs are characterized by longer circulation times due to lower protein adsorption, also known as the protein corona [27]. Thus, the surface charge of all produced liposomes was investigated, and all formulations displayed zeta potential values close to 0 mV (Table 1), which is expected considering the lipids composing the liposomes. No significant variations in the liposomes’ zeta potential were noticed, regardless of the technique used for the NPs production and GA encapsulation (*p* > 0.05).

Another important parameter to consider when developing DDS is the EE, which represents the drug’s loading efficiency. EE depends on the properties of both drug and NPs, including the drug’s molecular weight and solubility, NPs size, and drug-NPs chemical interactions, among others [32]. According to Table 1, the EE and LC values are influenced by the liposome production technique (*p* < 0.05) in the following sequence: reverse-phase evaporation > lipid film hydration = ethanol injection = ethanol permeabilization. Gomez et al. (2019) [33] reported similar findings when encapsulating the antibiotic vancomycin hydrochloride into liposomes using distinct techniques. Higher EE was obtained using reverse-phase evaporation, while lipid film hydration showed the lowest values. For this reason, reverse-phase evaporation was used to produce unloaded and GA-loaded liposomes in the following experiments.

### 3.2. Functionalization of Liposomes with Tf

**Figure 2:**
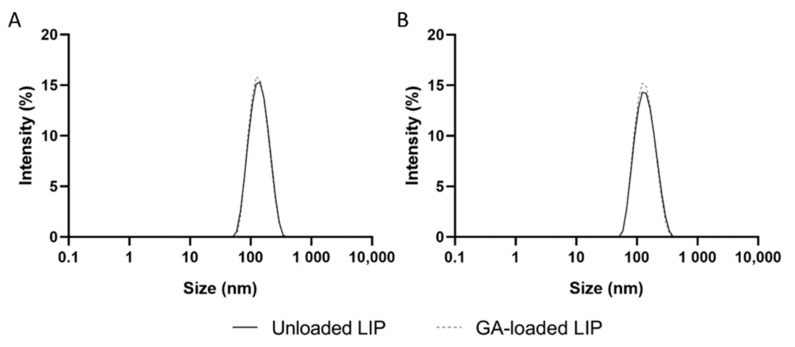
Size distribution by intensity of unloaded (black line) and GA-loaded liposomes (dashed gray line) (**A**) before and (**B**) after Tf functionalization.

The conjugation of specific ligands to the NPs’ surface has been widely employed to enhance their transport across the BBB [34]. An appropriate choice of the ligand is crucial to increasing the transport efficiency of the NPs, since the receptor must be expressed at the target site, in this case, at the BBB [35]. TfR is currently the most relevant target for enhanced drug delivery to the brain since it is present in a more significant amount in BBB’s capillary endothelium than in other organs [36]. TfR are also overexpressed in neuronal cells [37]. Because Tf is easily obtained from human sources in high abundance and low cost, it is the most studied targeted ligand for TfR-mediated brain delivery. Therefore, GA-loaded liposomes were produced by reverse-phase evaporation and functionalized with Tf, envisaging the dual-targeting of GA to the BBB and neuronal cells. EDC was used as a coupling agent to functionalize the NPs by activating the Tf’s carboxyl groups through a carbodiimide-coupling reaction. This allows the covalent coupling of activated Tf with DSPE-PEG2000 amine’s amino group. The physicochemical properties of unloaded and GA-loaded liposomes, before and after Tf functionalization, are presented in Table 2. The size distribution by intensity of all formulations is shown in Figure 2.

As shown in Table 2, the physicochemical properties of unloaded or GA-loaded liposomes were not affected by the functionalization with Tf (*p* > 0.05), with both formulations exhibiting mean sizes of 130 nm and zeta potential values close to 0 mV. Furthermore, all liposomal formulations were found to have a narrow size distribution (Figure 2), with PdI values lower than 0.2. No significant variations in the EE and LC were observed before and after the NPs’ functionalization (*p* > 0.05), implying that no GA was released during the process. This is in line with earlier research revealing that storing lipid-based NPs at 4 °C, the temperature at which the liposomes’ functionalization was executed, prevents drug leakage [38,39]. Table 2 further shows that the CE of GA-loaded liposomes is significantly lower than unloaded NPs (*p* < 0.05). This may be explained by the presence of GA molecules at the liposomes’ surface, which may hamper the covalent coupling between activated Tf and DSPE-PEG2000 amine. This hypothesis is supported by Andrade et al. (2019), who demonstrated that the GA establishes electrostatic interactions with the phospholipids’ polar heads [40]. The CE values in Table 2 translate into 119 ± 10 of Tf molecules per unloaded liposome and 86 ± 11 per GA-loaded liposome. Both values fall within the range of Tf molecules per NP needed to cross the BBB by RMT [41], thus ensuring the effectiveness of the developed systems in targeting and crossing the BBB. The obtained results confirm thus that the produced GA-loaded Tf-functionalized liposomes show appropriate physicochemical properties for brain delivery.

**Figure 3:**
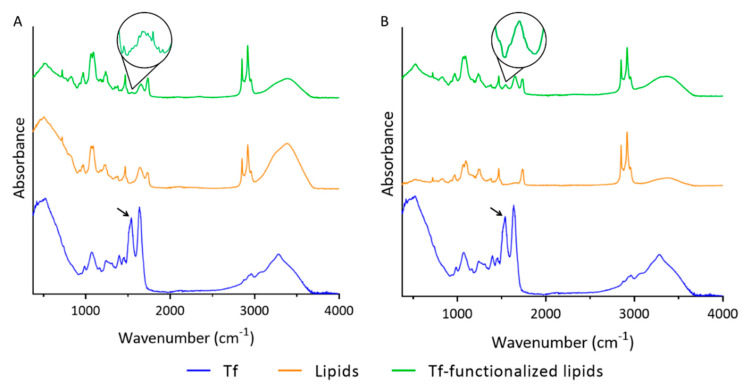
ATR-FTIR spectra of Tf (blue line), non-functionalized lipids (orange line), and Tf- functionalized lipids (green line) in the (**A**) absence and (**B**) presence of GA. Black arrow represents the amino II peak at 1540 cm^−1^.

ATR-FTIR was used to validate the lipids’ functionalization with Tf molecules. ATR-FTIR spectra of unloaded and GA-loaded liposomes, without and with Tf functionalization, are illustrated in Figure 3. For comparison, the ATR-FTIR spectrum of Tf is also provided.

Two major bands in the ATR-FTIR spectrum of Tf can be observed in Figure 3 (blue data). The peak at 1636 cm^−1^ is assigned to the amine I vibrations, while the band at 1540 cm^−1^ corresponds to amine II. These major peaks are ascribed to the C=O stretching and N-H bending vibrations, respectively, present in the peptide bonds between Tf’s amino acids [42]. In contrast to non-functionalized lipids (orange data), which lack the amine II peak, Tf-functionalized lipids (green data) present both peaks at the same wavenumber as Tf (blue data). Similar ATR-FTIR spectra were obtained in the presence of GA (Figure 3B). The presence of the amino II peak at 1540 cm^−1^ in the ATR-FTIR spectra of Tf-functionalized lipids in the absence and presence of GA (black arrow) can thus be assigned to the successful functionalization of the lipids with Tf.

### 3.3. Physical Stability of Liposomes at Storage Conditions

**Figure 4:**
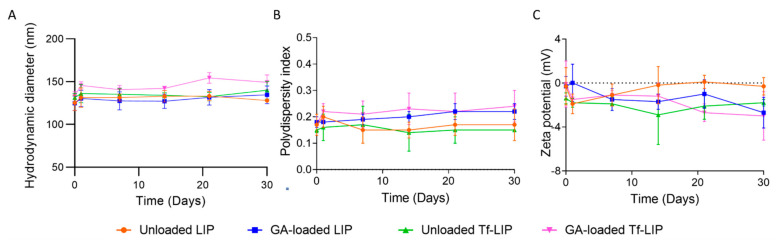
Physicochemical properties of unloaded (orange), GA-loaded (blue), Tf-functionalized (green), and GA-loaded Tf-functionalized liposomes (pink) in terms of (**A**) hydrodynamic diameter, (**B**) PdI, and (**C**) zeta potential, stored at 4 °C for 1 month.

One of the potential barriers to the use of liposomes in therapeutic applications is their lipids’ physical and chemical instability. The long-term storage of liposomes can cause phospholipids’ degradation, which can in turn induce changes in the liposomes’ permeability, structure, and drug retention. Storing liposomal formulations in the aqueous form is typically preferred over lyophilization and rehydration since these procedures result in a variation in the NPs’ size and drug leakage. Doxil^®^ and Onivyde^®^ are commercially available liposomal formulations stored in the fluid state [43]. Thus, the physical stability of the produced liposomal formulations was investigated at storage conditions (4 °C) for 1 month. The mean diameter, PdI, and zeta potential of the unloaded and GA-loaded liposomes, before and after Tf functionalization, were periodically recorded since variations in the liposomes’ physicochemical properties suggest changes in their structure [44]. The results are shown in Figure 4.

The findings presented in Figure 4A show that the mean size of unloaded and GA-loaded liposomes, before and after Tf functionalization, remained constant over the experiment (*p* > 0.05). Moreover, no significant variations in the PdI and zeta potential of all formulations were detected over 1 month at 4 °C (*p* > 0.05). The visual observation of the samples revealed no signs of phase separation, flocculation, or creaming. According to these results, all formulations remained stable for 1 month under storage conditions, regardless of GA encapsulation or liposomes’ functionalization. Since neutral NPs are more likely to aggregate than charged ones due to the absence of electrostatic repulsions between NPs, different approaches have been implemented to reduce the propensity of neutral NPs to aggregate, such as the modification of NPs’ surface with polymers. The presence of PEG in liposomes’ composition is known to improve the colloidal stability of neutral liposomes due to steric stabilization [45,46]. More specifically, PEG polymers create a highly hydrated layer that sterically inhibits electrostatic and hydrophobic interactions between liposomes, thus avoiding vesicle aggregation [9,47].

The stability of GA under storage conditions was also investigated. The absorbance of GA was monitored over 1 month at 4 °C, and no significant variations were observed (*p* > 0.05) (Figure S1 of Supplementary Material). These findings allowed us to conclude that no alterations in the GA’s chemical structure occurred. Furthermore, liposomes are known to shield molecules from degradation, hence preserving their therapeutic activity [48]. Therefore, it is expected that encapsulated GA maintains its bioactivity after storage.

### 3.4. In Vitro Release of GA from Tf-Functionalized Liposomes

**Figure 5:**
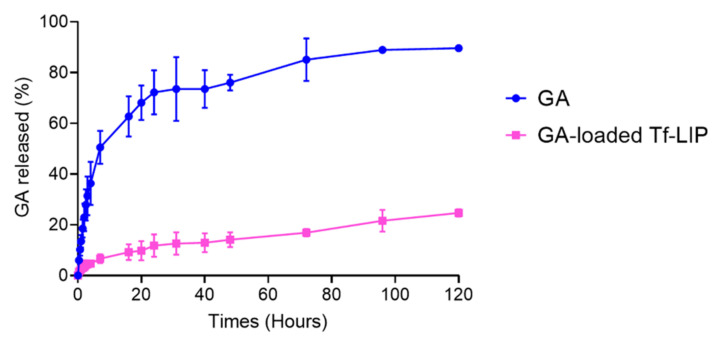
In vitro release profile of GA from Tf-functionalized liposomes (pink data) at 37 °C in PBS (pH 7.4, 10 mM) for 120 h. Blue data shows the in vitro release profile of free GA.

The efficacy of a DDS is significantly impacted by the drug’s release profile. It is desired that drug release from NPs occurs in a sustained manner so that the drug concentration in plasma can be maintained at therapeutic levels for prolonged periods. This way, it is possible to obtain optimal drug therapeutic efficacy while reducing its side effects [49]. Therefore, the in vitro release of GA from the Tf-functionalized liposomes was investigated under simulated physiological conditions (37 °C, PBS, pH 7.4, 10 mM) using the dialysis bag technique. The in vitro release of free GA was also attained for comparison purposes. The results are displayed in Figure 5.

As shown in Figure 5, the in vitro release profile of GA from the Tf-functionalized liposomes (pink data) follows a sustained tendency, unlike free GA (blue data). While 25 ± 2% of GA was released from the NPs after 120 h, free GA reached a plateau after 96 h, with 90 ± 1% of molecules being released in this period (*p* < 0.05). About 12 ± 4% of GA was released from the Tf-functionalized liposomes in the first 24 h. The remaining 13% were released over the following 4 days. This initial burst release followed by a slower and controlled release was previously observed [50], and it has been attributed to the desorption of adsorbed molecules at the NP’s surface [51]. This particular biphasic release pattern allows most of the compound to reach the target site [48]. Similar in vitro drug release profiles from liposomes have previously been reported [1,52].

Since GA has limited solubility in water, most GA molecules are expected to remain within the phospholipid bilayer of the Tf-functionalized liposomes instead of being released into the release medium. This hypothesis is supported by previous evidence [40], which demonstrated that GA has a higher affinity for the liposomes’ bilayer than for the aqueous medium. The same authors also verified that GA is preferentially located in the hydrophobic region of the lipid membrane [40]. In addition to the GA’s properties, the presence of PEG and Tf molecules at the liposomes’ surface also hinders the drug diffusion to the release medium [53,54]. Once NPs have reached the target site, it is expectable that the remaining encapsulated GA will be released in a controlled and sustained manner as the liposomes are degraded [48]. The prepared DDS thus presents an appropriate release profile for the brain delivery of GA.

### 3.5. Anti-Amyloidogenic Activity of GA-Loaded Liposomes Functionalized with Transferrin

#### 3.5.1. Inhibition of Aβ Fibril Formation

The therapeutic potential of the developed brain-targeted DDS to prevent AD was investigated. For this purpose, the ability of GA-loaded Tf-functionalized liposomes to inhibit Aβ_1-42_ aggregation was assessed using a ThT fluorescence assay, a widely employed protocol used to monitor amyloid fibril formation. This experiment is based on the ThT’s ability to emit fluorescence when binding to amyloid fibrils. Scientists established a direct relationship between ThT fluorescence intensity and the amount of amyloid fibrils [25].

**Figure 6:**
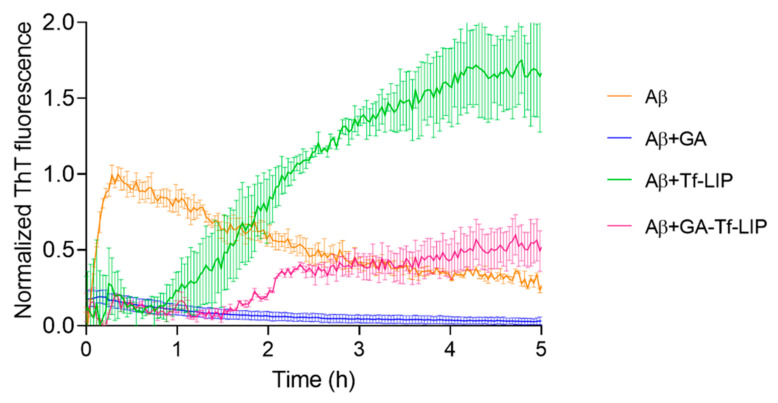
Normalized ThT fluorescence of Aβ_1-42_ (orange data) as a function of time (h) upon incubation with GA (blue data), Tf-functionalized liposomes (green data), and GA-loaded Tf-functionalized liposomes (pink data).

In this assay, Aβ monomers were incubated with GA, unloaded, and GA-loaded Tf-functionalized liposomes for 5 h at 37 °C. ThT fluorescence intensities were continuously recorded and are displayed in Figure 6. Equations (6) and (7) were applied to the obtained ThT data to calculate the kinetic parameters of each group, which are shown in Table 3.

Figure 6 depicts typical sigmoidal curves for the kinetics of Aβ in the absence (orange data) and presence of unloaded (green data) and GA-loaded Tf-functionalized liposomes (pink data). At the beginning of each curve, the fluorescence emitted by ThT is low, suggesting the presence of few Aβ fibrils in the samples. Aβ monomers gather to form oligomers in this phase, frequently referred to as the lag phase. In the next phase, termed the elongation phase, oligomers group together to form Aβ fibrils. The formation of the fibrils is found to cause a progressive rise in the ThT fluorescence intensity in this phase, followed by the stationary phase, which is marked by a plateau after all the Aβ peptides have been assembled into fibrils.

As shown in Figure 6, the Aβ fibrillation kinetic time course (orange data) was modified considerably by adding GA to the Aβ monomers (blue data). No discernible rise in the ThT fluorescence signal was seen (*p* > 0.05) (blue data), suggesting that GA entirely prevents the formation of Aβ fibrils. Instead, after 5 h of incubation, GA caused a slight reduction in the ThT signal (*p* < 0.05). This can be due to the GA’s ability to disrupt existing fibrils from the examined sample. Similar outcomes have previously been reported [25].

Figure 6 further shows that the formation of Aβ fibrils was dramatically influenced by unloaded Tf-functionalized liposomes (green data). Table 3 reveals that the empty liposomes dramatically extended the lag phase duration compared to Aβ from 0.015 ± 0.002 h (approximately 1 min) to 0.9 ± 0.4 h (about 54 min) (*p* < 0.05), suggesting that the presence of NPs inhibits the conversion of Aβ monomers to oligomers. Unloaded Tf-functionalized liposomes also appear to impede the transition from oligomers to fibrils since they dramatically decreased the elongation rate compared to untreated Aβ from 22.7 ± 0.6 to 1.9 ± 0.1 h^−1^ (*p* < 0.05). As a result, the time needed to reach 50% of the maximum ThT fluorescence intensity increased 20-fold. However, the maximal ThT fluorescence almost doubled in the presence of unloaded Tf-functionalized liposomes than in the absence (*p* < 0.05), suggesting that NPs increase the number of fibrils formed. The results are consistent with other studies showing that lipid-based NPs, including liposomes, promote Aβ fibril formation [39,55].

The Aβ’s fibrillation kinetics were also impacted by the presence of GA-loaded Tf-functionalized liposomes (pink data), as depicted in Figure 6. Similar to unloaded NPs, GA-loaded NPs also substantially lengthened the lag phase time compared to untreated Aβ from 0.015 ± 0.002 h (approximately 1 min) to 1.4 ± 0.03 h (about 84 min) (*p* < 0.05), suggesting that the DDS slows the conversion of Aβ monomers to oligomers. Additionally, a substantial reduction in elongation rate was seen in comparison to untreated Aβ (orange data) from 22.7 ± 0.6 h^−1^ to 4.9 ± 3.4 h^−1^ (*p* < 0.05), which lengthened the time required to attain half of the maximal ThT fluorescence intensity by 22 times (*p* < 0.05). These results indicate that the developed DDS inhibits the Aβ oligomers to fibrils transition. According to Table 3, GA-loaded Tf-functionalized liposomes also dramatically lowered the maximum ThT fluorescence intensity compared to untreated Aβ from 0.9 ± 0.1 to 0.5 ± 0.2 (*p* < 0.05), implying a reduction of around 56% in the number of fibrils formed. Moreover, the presence of GA in Tf-functionalized NPs significantly decreased the ThT signal compared to unloaded NPs from 1.7 ± 0.3 to 0.5 ± 0.2 (*p* < 0.05), suggesting that the natural compound released so far is enough to prevent the nucleation effect of the empty NPs. Thus, the presented results validate the therapeutic potential of the developed brain-targeted DDS to delay the onset of AD.

**Figure 7:**
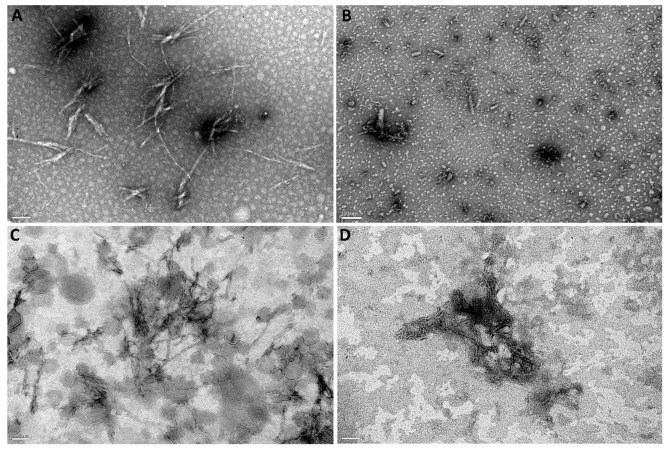
TEM images of (**A**) Aβ_1-42_ incubated with (**B**) GA, (**C**) Tf-functionalized liposomes, and (**D**) GA-loaded Tf-functionalized liposomes for 5 h at 37 °C. Scale bars correspond to 100 nm.

The ability of the developed brain-targeted DDS to prevent fibril formation was also investigated by TEM. This widely employed technique offers detailed information on the amyloid aggregates’ structure, size distribution, and structural characteristics. Figure 7 shows the TEM images of Aβ incubated with GA, unloaded, and GA-loaded NPs for 5 h at 37 °C. After incubation, untreated Aβ formed several thin and long fibrils (Figure 7A). The quantitative analysis of the Aβ fibrils’ morphological features was investigated in terms of length and thickness. Untreated Aβ had lengths ranging from 70 nm to 480 nm, and thickness varying between 2 nm and 10 nm, which is in agreement with previous studies [56]. However, the presence of GA significantly prevented fibril formation as fewer Aβ fibrils were present in the sample (Figure 7B). Instead, numerous small structures are observed, confirming the GA’s anti-aggregation properties. As expected by ThT data, Aβ fibrils were also seen when Aβ was incubated with unloaded (Figure 7C) and GA-loaded NPs (Figure 7D). These fibrils appear shorter (maximum 370 nm) compared to untreated Aβ. No significant differences in the fibrils’ thickness were noticed. However, fewer fibrils were observed in the presence of GA-loaded Tf-functionalized liposomes (Figure 7D) compared to untreated Aβ (Figure 7A), thus validating the DDS’s ability to prevent fibril formation.

#### 3.5.2. Disaggregation of Mature Aβ Fibrils

**Figure 8:**
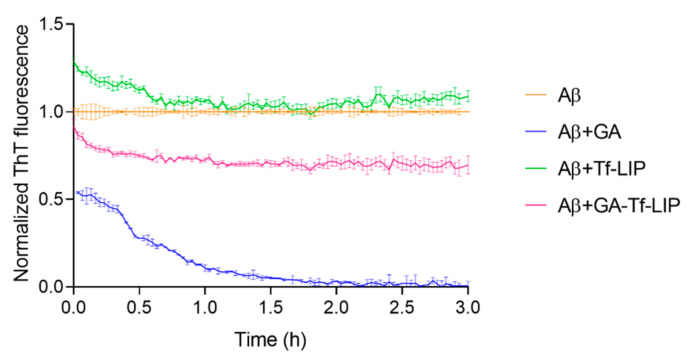
Normalized ThT fluorescence of mature Aβ_1-42_ fibrils (orange data) as a function of time (h) upon incubation with GA (blue data), Tf-functionalized liposomes (green data), and GA-loaded Tf-functionalized liposomes (pink data).

The therapeutic potential of the developed brain-targeted DDS to treat AD was studied. For this purpose, the capability of GA-loaded Tf-functionalized liposomes to disaggregate mature fibrils was evaluated using a ThT fluorescence assay. In this experiment, Aβ fibrils were prepared by incubating Aβ monomers at 37 °C for 2h. After that, Aβ fibrils were incubated with GA, unloaded, and GA-loaded Tf-functionalized liposomes for 3 h at 37 °C while the ThT fluorescence signals were recorded. The results are displayed in Figure 8. Table 4 presents the amount of Aβ fibrils after their incubation with GA, unloaded, and GA-loaded Tf-functionalized liposomes at the beginning and end of the experiment.

The Aβ fibrils’ content was significantly impacted by whether GA (blue data), unloaded (green data), or GA-loaded Tf-functionalized liposomes (pink data) were added, as shown in Figure 8. The ThT fluorescence signal was immediately reduced after adding GA to the fibrils by around 46% (*p* < 0.05). As shown in Table 4, ThT fluorescence gradually decays over time until reaching a 99% drop after 3 h of incubation. These results support earlier findings that GA can disrupt Aβ fibrils.

Additionally, Figure 8 demonstrates that the addition of unloaded NPs (green data) considerably boosted the ThT fluorescence signal in comparison to untreated Aβ fibrils (orange data) (*p* < 0.05). According to Table 4, unloaded Tf-functionalized liposomes immediately raised the fibril content by around 29% (*p* < 0.05), which decreased by 20% after 3 h of incubation at 37 °C (*p* < 0.05). This may be related to the aggregation of some prefibrillar species present in the sample. Adding GA to the NPs (pink data) significantly contradicted the unloaded NPs’ amyloidogenic action (green data) (*p* < 0.05). No significant variations in fibril content were observed immediately after adding GA-loaded Tf-functionalized liposomes to Aβ fibrils (*p* > 0.05). However, after 3 h of incubation, the ThT fluorescence intensity dropped by about 30% compared to untreated Aβ fibrils, (*p* < 0.05) and 39% compared to unloaded NPs (*p* < 0.05). The data, therefore, support the therapeutic potential of the produced brain-targeted DDS to treat AD.

**Figure 9:**
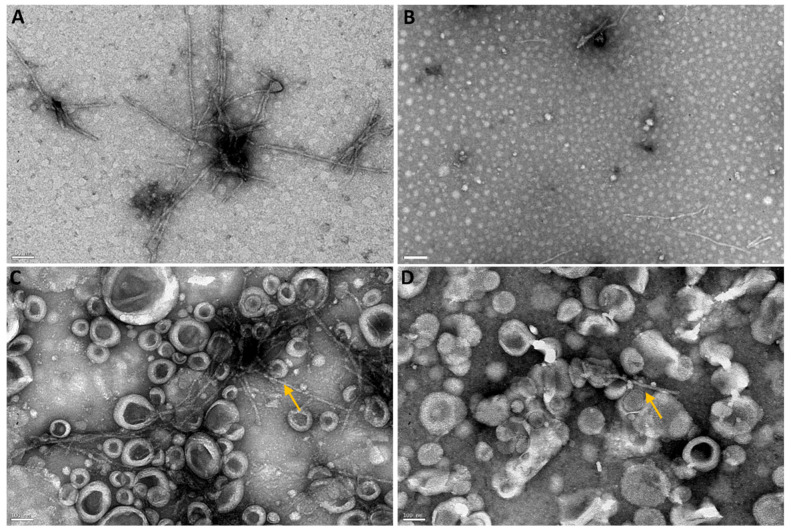
TEM images of (**A**) Aβ fibrils incubated with (**B**) GA, (**C**) Tf-functionalized liposomes, or (**D**) GA-loaded Tf-functionalized liposomes for 3 h at 37 °C. Yellow arrows show Aβ fibrils. Scale bars are 100 nm.

The Aβ’s morphology after incubating fibrils with GA, unloaded, and GA-loaded Tf-functionalized liposomes for 3 h at 37 °C was examined by TEM (Figure 9). Numerous slender and lengthy fibrils were seen when Aβ fibrils were incubated alone (Figure 9A). The observed fibrils present lengths ranging between 180 and 570 nm, and thickness varying between 7 and 18 nm. However, the presence of GA significantly reduced the number of fibrils (Figure 9B). The structures observed in Figure 9B appear shorter than untreated Aβ fibrils (Figure 9A), thus validating the GA’s ability to disaggregate preformed Aβ fibrils. From Figure 9C,D, it is possible to identify Aβ fibrils (yellow arrows) in the presence of unloaded and GA-loaded liposomes, respectively. No major differences in the fibrils’ morphology were observed compared to untreated Aβ fibrils. However, fewer fibrils were noticed in the presence of GA-loaded Tf-functionalized liposomes than unloaded liposomes.

## 4. Conclusions

In this work, Tf-functionalized liposomes for the delivery of GA were designed. Firstly, GA-loaded liposomes were prepared using distinct techniques to find the formulation with the most suitable physicochemical properties, including size, PdI, zeta potential, EE, and LC. The liposomal formulation obtained using the reverse-phase evaporation technique was revealed to be the most appropriate in terms of NPs’ physicochemical characteristics for brain delivery purposes. Then, the GA-loaded liposomes’ surface was functionalized with Tf to improve their passage across the BBB. ATR-FTIR spectroscopy confirmed the successful functionalization of the lipids with Tf. Stability studies under storage conditions proved the colloidal stability of the nanosystem over 1 month. Furthermore, the in vitro release of GA from Tf-functionalized liposomes was slow and sustained. Afterward, the neuroprotective effect of the developed DDS was investigated using an in vitro model of AD. Studies were conducted to evaluate the ability of the nanosystem to interact with Aβ_1-42_ monomers and fibrils. The results demonstrated that GA-loaded Tf-functionalized liposomes could inhibit Aβ aggregation and fibrillation and disrupt preformed fibrils. The established GA-loaded DDS could be a promising approach for AD therapy. Nevertheless, further studies with animal models of AD are warranted to validate the therapeutic efficacy of this novel liposomal formulation.

## Figures and Tables

**Table 1:** Physicochemical properties of unloaded and GA-loaded liposomes, including hydrodynamic diameter, PdI, zeta potential, EE, and LC.

Technique	Liposomes’ Formulation	Size (nm)	PdI	Zeta Potential (mV)	EE (%)	LC (%)
Lipid film hydration	Unloaded LIP	117 ± 6	0.17 ± 0.03	−2.3 ± 0.6	-	-
	GA-loaded LIP	112 ± 4	0.16 ± 0.02	−2.2 ± 0.1	12 ± 2	0.7 ± 0.1
Reverse-phase evaporation	Unloaded LIP	129 ± 10	0.17 ± 0.04	−0.7 ± 1.0	-	-
	GA-loaded LIP	126 ± 10	0.18 ± 0.02	−0.4 ± 0.8	29 ± 3	1.7 ± 0.2
Ethanol injection	Unloaded LIP	163 ± 4	0.20 ± 0.03	−1.9 ± 0.5	-	-
	GA-loaded LIP	150 ± 12	0.21 ± 0.07	−1.2 ± 0.4	11 ± 4	0.6 ± 0.2
Ethanol permeabilization	Unloaded LIP	145 ± 2	0.19 ± 0.01	−0.8 ± 0.4	-	-
	GA-loaded LIP	147 ± 5	0.16 ± 0.03	−1.7 ± 0.1	15 ± 4	0.9 ± 0.3

**Table 2:** Physicochemical properties of unloaded and GA-loaded liposomes, without and with Tf functionalization. * Implies a statistical difference between GA-loaded liposomes before and after Tf functionalization (*p* < 0.05).

Formulation	Size (nm)	PdI	Zeta Potential (mV)	EE (%)	LC (%)	CE (%)
Unloaded LIP	129 ± 10	0.17 ± 0.04	−0.7 ± 1.0	-	-	-
Unloaded Tf-LIP	132 ± 4	0.15 ± 0.02	−1.3 ± 0.5	-	-	35 ± 3
GA-loaded LIP	126 ± 10	0.18 ± 0.02	−0.4 ± 0.8	29 ± 3	1.7 ± 0.2	-
GA-loaded Tf-LIP	131 ± 9	0.16 ± 0.02	−0.8 ± 1.0	31 ± 6	1.8 ± 0.4	26 ± 3 *

**Table 3:** Kinetic parameters were calculated by fitting Equations (6) and (7) to the ThT data. * Indicates a statistical difference compared to Aβ (*p* < 0.05). ^#^ Indicates a statistical difference between unloaded and GA-loaded Tf-functionalized liposomes (*p* < 0.05).

Kinetic Parameters	Aβ	Aβ + Tf-LIP	Aβ + GA-Tf-LIP
tlag (h)	0.015 ± 0.002	0.9 ± 0.4 *	1.4 ± 0.03 *
t_1/2_ (h)	0.10 ± 0.01	2.0 ± 0.5 *	2.2 ± 0.3 *
k (h^−1^)	22.7 ± 0.6	1.9 ± 0.1 *	4.9 ± 3.4 *
Max. Fluorescence	0.9 ± 0.1	1.7 ± 0.3 *	0.5 ± 0.2 *^,#^

**Table 4:** Amount of Aβ_1-42_ fibrils upon their incubation with GA, unloaded, and GA-loaded Tf-functionalized liposomes for 3 h at 37 °C. * Indicates a statistical difference compared to untreated Aβ fibrils (*p* < 0.05). ^#^ Indicates a statistical difference between the beginning and end of the experiment (*p* < 0.05). ^§^ Indicates a statistical difference between unloaded and GA-loaded Tf-liposomes (*p* < 0.05).

Aβ Fibril Content (%)			
Time (h)	Aβ + GA	Aβ + Tf-LIP	Aβ + GA-Tf-LIP
0	54 ± 5 *	129 ± 2 *	92 ± 4 ^§^
3	1 ± 1 *^,#^	109 ± 3 ^#^	70 ± 5 *^,#,§^

## Supplementary Materials

The following supporting information can be downloaded at: https://www.mdpi.com/article/10.3390/pharmaceutics14102163/s1, Figure S1: Absorbance of GA (150 µM) in PBS (pH 7.4, 10 mM) stored in the dark at 4 °C for 1 month. Measurements were performed at 258 nm using a Synergy™ 2 Multi-Mode Microplate Reader (BioTek Instruments, Winooski, VT, USA), with a well volume of 300 µL.
