# Supplementary material for: Transferrin-Functionalized Liposomes for the Delivery of Gallic Acid: A Therapeutic Approach for Alzheimer’s Disease
Source: Pharmaceutics. 2022 Oct 11;14(10):2163. doi: 10.3390/pharmaceutics14102163 (PMC9609970; doi:10.3390/pharmaceutics14102163)
Supplement: Supplementary file 1 [file pharmaceutics-14-02163-s001.zip › pharmaceutics-1916657-supplementary.pdf]

Supplementary Material

Transferrin-Functionalized Liposomes for the Delivery of Gallic Acid: A Therapeutic Approach for Alzheimer's Disease

Stéphanie Andrade, Joana A. Loureiro, and Maria C. Pereira

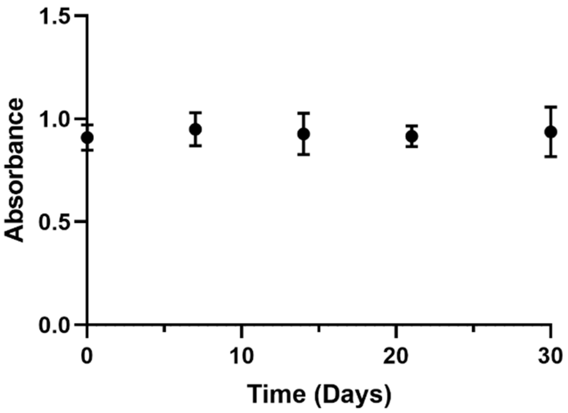

Figure S1. Absorbance of GA (150 μM) in PBS (pH 7.4, 10 mM) stored in the dark at 4 °C for 1 month. Measurements were performed at 258 nm using a Synergy™ 2 Multi-Mode Microplate Reader (BioTek Instruments, Winooski, VT, USA), with a well volume of 300 μL.
